# Case report of paroxysmal dystonia in a child with KBG syndrome: Expansion of the phenotype and utility of whole exome sequencing

**DOI:** 10.1097/MD.0000000000043631

**Published:** 2025-08-01

**Authors:** Christina R. Dantam, Elizabeth Wilkes, Holly N. Summers, Colleen A. Morris, Rooman F. Ahad

**Affiliations:** a Kirk Kerkorian School of Medicine, University of Nevada, Las Vegas, Las Vegas, NV; b Grant a Gift Autism Foundation – Ackerman Center in alliance with UNLV Kirk Kerkorian School of Medicine, Las Vegas, NV; c Department of Pediatrics, Kirk Kerkorian School of Medicine at UNLV, Las Vegas, NV.

**Keywords:** KBG syndrome, paroxysmal dystonia, whole exome sequencing

## Abstract

**Rationale:**

KBG syndrome is a rare, autosomal dominant neurodevelopmental disorder characterized by developmental delay, macrodontia, distinctive facial features, and a range of systemic manifestations.

**Patient concerns:**

We report a pediatric patient with a history of global developmental delay, autism spectrum disorder, sensorineural hearing loss, and spastic diplegia who developed episodic, unilateral dystonic spells beginning at age 7, leading to impaired mobility.

**Diagnoses:**

Initial genetic testing revealed a maternally inherited 3p26 duplication, which did not fully account for the patient’s clinical presentation. Whole exome sequencing (WES) was subsequently performed and identified a pathogenic frameshift mutation in *ANKRD11*, confirming a diagnosis of KBG syndrome. Additional genetic variants were found in *CDH23*, potentially explaining the patient’s profound hearing loss.

**Interventions:**

After receiving a diagnosis, the patient received multidisciplinary care including intensive speech, occupational, physical, applied behavior analysis therapies, and educational planning to address his neurodevelopmental needs.

**Outcomes:**

WES established a unifying diagnosis that better accounted for the patient’s constellation of findings. Recognition of KBG syndrome facilitated appropriate medical, rehabilitative, and educational interventions. The presence of paroxysmal dystonia, previously unrecognized in KBG syndrome, adds to the expanding phenotypic spectrum.

**Lessons:**

This case underscores the diagnostic value of WES in patients with complex neurodevelopmental presentations and unexplained movement disorders. Our findings support the inclusion of *ANKRD11* in the differential for pediatric dystonia and suggest a potential, previously underrecognized neurologic feature of KBG syndrome. Broader access to genomic diagnostics may reduce the diagnostic odyssey for similar patients and inform more targeted care strategies.

## 1. Introduction

Diagnostic accuracy in children with neurodevelopmental disorders often improves over time due to the evolution of the individual’s phenotype as well as improvements in laboratory technology. We had the opportunity to reevaluate a child who initially presented at 17 months with microcephaly, short stature, spastic diplegia, developmental delay, hearing impairment, and autism who was found to have a maternally inherited 1.63Mb 3p26 duplication involving genes *CNTN6*, *CNTN4*, and *CNTN4-AS2.* At age 7 years, his phenotype was notable for additional findings: dysmorphic features including large central incisors, ADHD, and episodes of dystonia of the right side that interfered with ambulation. Exome sequencing revealed a pathogenic mutation in *ANKRD11* (ankyrin repeat domain 11), diagnostic of KBG syndrome.

KBG syndrome, first described in 1975, is caused by loss of function heterozygous mutation or deletion of *ANKRD11*.^[1]^ The gene is an important chromatin regulator in neurogenesis. KBG syndrome is characterized by macrodontia of the central incisors, dysmorphic facial features, short stature, developmental delay, intellectual disability, autism, ADHD, seizures, and hearing loss.^[1–3]^ While the exact prevalence of KBG syndrome has not been established, over 300 cases have been reported.^[2]^ This report hopes to expand on the clinical features associated with KBG syndrome and look at the potential benefits of exome sequencing to better identify symptoms in patients with unexplained clinical findings.

## 2. Patient concerns

We report a patient who presented to the pediatric neurology clinic with episodic tremors with dystonia of the right side, a finding not previously reported in KBG syndrome. These dystonic spells were different from his baseline spastic diplegia.

## 3. Case report

Informed consent was obtained from the patient’s parents for the purpose of publication. Our male patient was born via Cesarean section at 37 weeks gestation but was kept in the NICU for 1 week due to respiratory distress and feeding problems. Growth parameters at birth were as follows: OFC: 33cm (9%) BW: 2689gm (8%) BL: 43cm (<3%). He failed the newborn hearing screen and had unilateral postaxial polydactyly. The family history was positive for 1 previous spontaneous abortion to this couple, in addition to multiple spontaneous abortions and 2 stillbirths to the maternal grandmother. Genetics evaluation at 16 months was positive for microcephaly, hypertonia, and spastic diplegia. He had sensorineural hearing loss, fleeting eye contact, rocking, and stereotypic movements. Chromosome microarray showed a 3p26 duplication, inherited from the mother: arr[hg19] 3p26.3p26.2 (1311,741-2940,382) × 3 (mat). Fragile X studies were negative. An MRI obtained at 18 months was normal.

Neurological evaluation at age 2 showed a child with delays in speech and gross motor skills, sensorineural hearing loss, and stereotypic movements involving upper and lower limbs. The patient began walking and did not have a follow-up until much later. The patient was followed by ophthalmology and was found to have hyperopia and astigmatism along with accommodative esotropia. His funduscopic exam was normal. A cochlear implant was placed at the age of 3 years.

At 7 years old, he was seen by the developmental pediatrics team for hyperactivity and impulsive behaviors. During that visit, his mother described spells that caused stiffness in his right leg and arm. These spells were different from his baseline spastic diplegia. He was referred back to neurology due to diagnostic uncertainty between partial onset seizures and a movement disorder. These episodes caused impairment in mobility and loss of function. The spells were peculiar as they involved some tremors and joint immobility. Episodes occurred twice that year and lasted several weeks, with full resolution of symptoms in between. He would retain consciousness during these events. On neurological examination, the patient was alert and awake but non-verbal at baseline. Although he managed to stand for a few seconds at a time, his legs shook while upright. During ambulation, he exhibited dystonic posturing affecting his right side. At times, he would resort to crawling. The spells did not seem to cause him pain. He remained alert and interactive throughout. Both episodes lasted a few weeks, occurred 6 months apart, and resolved without intervention or treatment. A routine EEG was performed and yielded normal. The patient was also noted to have difficulty with falling and staying asleep. He was quite hyperactive in the evenings. The patient was treated with low dose clonidine and hydroxyzine for sleep. At 7 years old, his height and weight were at the 2nd percentile, and his head circumference was at < 2nd percentile. He had a low anterior hairline, triangular-shaped face, flattened occiput, large ears, large central incisors, flat feet, and fifth finger clinodactyly. Previous genetic studies (3p26 duplication) did not explain all of his clinical findings. Therefore, oral saliva swabs were taken from the patient and mother to perform whole exome sequencing (WES) through Ambry Genetics. A pathogenic mutation was identified in *ANKRD11,* diagnostic of KBG syndrome (*ANKRD11* c.3770_3771delAA) (p.K1257Rfs*25). This deletion of 2 nucleotides in exon 9 causes a translational frameshift, resulting in a premature stop codon and subsequent loss of function. This genetic alteration has been documented in other individuals diagnosed with KBG syndrome.^[4]^ Also of interest, he was found to have 2 heterozygous variants of *CDH23* (c.3179G > C (p.R1060P) and c.4210-2A > G), which were classified as variants of uncertain significance associated with Usher syndrome type 1- *CDH23* related deafness. Exome sequencing for the mother showed a variant of unknown significance on CDH23 (c.3179G > C) (p.R1060P). The child’s unaffected mother was heterozygous for this alteration. Specific site analysis of the CDH23 c4210-2A > G variant of uncertain significance revealed that the unaffected mother is negative for this alteration. Genetic testing for the father was not obtained as the specimen was inadequate. After obtaining written consent from the parent to share WES data, an ascension number for the results was provided: 23-421615.

Upon reviewing exome sequencing results, further studies included a CT scan of the head that was normal. Bone age was delayed, showing 6-years-old at the chronological age of 8 years. His scoliosis survey showed mild curvature of the thoracolumbar spine with concavity to the right. No costovertebral anomalies were noted.

Neuropsychological testing at 17 months of age indicated significant delays in cognitive, adaptive, language, and motor development (Table 1).^[5–9]^ He also met criteria for autism spectrum disorder at 17 months and 4 years of age as evidenced by deficits in social reciprocity and repetitive behaviors beyond delays known to be associated with his genetic syndrome (only 3p26.23 duplication known at that time) (Table 1).^[10,11]^ After receiving his diagnosis, the patient received intensive speech, occupational, physical, and applied behavior analysis therapies. Brief partial screening repeated at age 4 indicated he remained developmentally delayed and non-speaking, although he had developed some non-verbal communication strategies (Table 1).^[8,10]^ Comprehensive testing at age 8 reflected a similar neurodevelopmental profile. He demonstrated severe delays in cognitive and adaptive development and ongoing symptoms associated with autism beyond his recently identified KBG syndrome (Table 1).^[6,7,9,11]^ The child is currently in the process of obtaining services at school to help support his educational needs as he is also non-verbal and has hearing loss. It is important to note that out-of-age range testing measures were used to determine an approximate age equivalence (Age Eq.) due to the patient’s level of cognitive impairment.

**Table 1:** Neuropsychological evaluation results.

Instrument	Age 17-mo	Age 4-yr (partial testing)	Age 8-yr
Bayley-III^[5]^ (17-mo) Bayley-4^[6]^ (8-yr)			
Cognitive	**55**; significant delay	–	–
	Age Eq. 7 mo	–	Age Eq. 17 mo
Language	**50;** significant delay	–	–
Receptive language	Age Eq. 2 mo	–	–
Expressive language	Age Eq. 5 mo	–	–
Motor	**46**; significant delay	–	–
Fine motor	Age Eq. 7 mo	–	–
Gross motor	Age Eq. 7 mo	–	–
Developmental profile-4^[7]^			
Cognitive	–	–	**40**; Age Eq. 1:4–1:5
Physical	–	–	**40**; Age Eq. 1:8–1:9
Adaptive	–	–	**40**; Age Eq. 1:8–1:9
Social-emotional	–	–	**40**; Age Eq. 1:0–1:1
Communication	–	–	**40**; Age Eq. 0:10–0:11
Vineland-II^[8]^ (17-mo and 4-yr) Vineland-3^[9]^ (8-yr)			
Communication	**52** (<1st percentile)	–	**36** (<1st percentile)
Daily living skills	**75** (5th percentile)	**59** (<1st percentile)	**56** (<1st percentile)
Socialization	**63** (1st percentile)	–	**44** (<1st percentile)
Motor skills	**58** (<1st percentile)	–	**54** (<1st percentile)
Childhood autism rating scale—2 standard version^[10]^			
Severity group	–	Mild-to-moderate	–
ADOS-2^[11]^ Toddler module (17-mo) Module 1 (8-yr)			
Social affect total	13	–	19
Restricted and repetitive behavior	8	–	6
Overall total ASD-related symptoms	21; moderate–severe	–	25; high

The results are listed as standard scores (bold) compared to same-aged peers. Due to significant intellectual impairment, some measures were out of the patient’s age range, and age-equivalence estimates are only provided in the table. Some publishers updated instruments; thus, different versions were used across time, as indicated. The neuropsychological assessment instruments used in the table above to evaluate the patient are supported by references^[5–11]^ of the reference list.

ADOS = autism diagnostic observation schedule, ASD = autism spectrum disorder.

## 4. Discussion

Our patient’s unique constellation of findings of microcephaly, autism, short stature, spastic diplegia, deafness, and dysmorphic features in conjunction with his transient right-sided dystonia were not sufficiently explained by the chromosome 3p duplication. This prompted exome sequencing, which identified a pathogenic mutation in the *ANKRD11* gene, diagnostic of KBG syndrome. KBG syndrome is characterized by a diverse clinical spectrum. In our patient, the phenotypic findings of dysmorphic facial features, short stature, hearing loss, and neurodevelopmental anomalies align with this diagnosis. However, his unilateral transient dystonia remains unexplained by the established features of KBG syndrome. We propose that the presence of paroxysmal dystonic spells in our patient expands the clinical picture of KBG syndrome, suggesting that this condition may present with a broader range of phenotypic manifestations than previously recognized. Movement disorders have rarely been reported in KBG syndrome. Donnellan et al (2024) reported a child who presented with a hyperkinetic movement disorder that evolved to choreoathetosis of the limbs and head with epileptic encephalopathy.^[12]^ Magistrelli et al (2023) reported an adult with KBG syndrome who developed Parkinson disease.^[13]^ Both of these individuals had abnormalities on EEG and MRI, unlike our patient. Because our patient’s movement disorder could be classified as a focal dystonia, the *ANKRD11* gene should be added to the list of genes that can cause neurodevelopmental delay and transient movement disorders.^[14]^

There are 3 genetic variations that likely contribute to the patient’s phenotype: 3p26 duplication, *ANKRD11* mutation, and *CDH23* variants. Duplications of chromosome 3p that include contactin genes *CNTN6* and *CNTN4* have been reported to be associated with neurodevelopmental disorders. The duplications are often inherited, but approximately one-third of parents with the duplication have neurodevelopmental symptoms, such as a learning disability, ADHD, and bipolar disorder.^[15]^ In the present case, screening of the mother’s cognitive abilities with the WASI-II reflected low average intellectual functioning (16th percentile), with average verbal comprehension (27th percentile) and low average perceptual reasoning (13th percentile).^[16]^ In a published series of chromosomal microarrays performed for developmental delay, 0.1% to 0.2% of samples have duplications in this region.^[17,18]^ The clinical findings common in children with 3p duplications include developmental delay (75%), ADHD (38%), Autism (29%), and short stature (16%).^[17–21]^ Only 1 patient has been previously reported with sensorineural hearing loss, the severity of which is not mentioned.^[17]^ The size of the duplication is variable, with most reported individuals having duplication of all or part of *CNTN6*. The phenotype is variable, with autism more likely in individuals with larger duplications that include *CNTN4*.^[19]^ In 1 series, 3 of 13 individuals with 3p duplication had other pathogenic copy number variants that likely contributed to their phenotype.^[18]^ While it is likely that the 3p duplication is contributing to our patient’s phenotype, KBG syndrome accounts for most of his clinical findings.

KBG syndrome (MIM 148050) is a neurodevelopmental disorder caused by deletion (22% of cases) or sequence variants (78% of cases) of the *ANKRD11* gene. It is inherited in an autosomal dominant fashion, but 90% of the sequence variants occur *de novo*.^[2]^ The phenotype is variable and is characterized by neurodevelopmental problems, dysmorphic facial features, short stature, and hearing loss. The craniofacial features include macrodontia of the central incisors in 80%, triangular face, brachycephaly, synophrys, widely spaced eyes, broad or bushy eyebrows, prominent ears, prominent nasal bridge, bulbous nose, anteverted nares, long philtrum, and thin vermilion of the upper lip. The largest review to date included 340 cases, combining new reports and literature cases.^[2]^ Prevalence of phenotypic features from that report are as follows: developmental delay, 95%; motor delay, 70%; intellectual disability, 80% (mild/borderline in 58%, severe in 5%); ADHD, 46%; autism spectrum disorder, 33%; visual problems, 56% (82% of these are refractive errors); seizures, 34%; abnormal EEG, 32%; abnormal MRI, 51%; hearing loss, 50% (sensorineural, 23%; conductive, 67%; mixed 10%); congenital heart disease, usually VSD, 36%; cryptorchidism 44%; microcephaly (OFC < 3rd%), 25%; birth length < 3rd%, 21%; postnatal short stature (<10th%), 58%. Delayed bone age and costovertebral anomalies have also been reported.

Hearing loss in KBG syndrome was specifically evaluated by Rhamati et al (2023).^[22]^ In that series, conductive hearing loss was most common, present in 71%, and sensorineural hearing loss was present in 17%. Only 1 patient in their series had profound congenital hearing loss treated with a cochlear implant. Our patient has profound sensorineural hearing loss and also has 2 *CHD23* variants detected on exome sequencing. While the variants have been interpreted as being of uncertain clinical significance, it is possible that these mutations, 1 a missense mutation and the other a mutation in a splice acceptor site, are pathogenic and account for the child’s deafness. Variants in the *CDH23* gene may cause either syndromic hearing loss (Usher syndrome type ID) or non-syndromic hearing loss (DFNB12).^[23]^

Our case demonstrates the utility of exome sequencing for diagnosing rare neurodevelopmental conditions in pediatric populations. We advocate for the integration of these technologies as a first- or second-line diagnostic tool for patients. Utilizing these technologies has numerous advantages, including the ability to detect a wide range of genetic variations, improving diagnostic yield, tailoring treatment plans to the patient’s genetic profile, saving costs by reducing the need for multiple tests, and shortening the diagnostic odyssey for patients.^[24,25]^ Potential harms include insurance discrimination, negative impacts on family dynamics, the financial burden of costs associated with additional testing, and loss of privacy.^[25,26]^ However, early use of WES in lieu of secondary genetic and metabolic testing could potentially result in significant cost savings, and parents are generally not reluctant or against their children undergoing WES despite insurance denying coverage for this service in 50% of cases.^[24]^ Currently, there is extensive variability in the use of WES in affected patients with cognitive anomalies, developmental delays, and intellectual disabilities. Establishing evidence-based protocols to define specific indications for its use is crucial for maximizing diagnostic yield and clinical utility.

## 5. Limitations

As this report describes a single patient, the generalizability of our findings regarding the potential association between KBG syndrome and paroxysmal dystonia is inherently limited. Although the patient’s clinical presentation supports an expansion of the KBG syndrome phenotype, causal conclusions are precluded by the coexistence of additional genetic variants, including a 3p26 duplication and *CDH23* mutations. Additionally, the absence of paternal genetic testing limits our ability to assess the potential contribution of undetected paternal variants.

## 6. Outcomes

WES established a unifying diagnosis that better accounted for the patient’s complex constellation of clinical findings, including developmental delay, autism, microcephaly, and dysmorphic features. The recognition of KBG syndrome provided a clearer diagnostic framework and guided more coordinated medical, rehabilitative, and educational planning. Notably, the patient’s episodes of paroxysmal, unilateral dystonia may represent a previously unrecognized manifestation of KBG syndrome. The resolution of these dystonic spells without intervention, alongside the absence of EEG or MRI abnormalities, suggests a self-limiting neurologic feature that may expand the known phenotypic spectrum of the disorder.

## 7. Conclusion

In conclusion, this case report highlights the complexities of diagnosing rare genetic syndromes, such as KBG syndrome, associated with the *ANKRD11* gene abnormality. Our patient first presented with an unknown neurodevelopmental disorder. Microarray testing found a 3p26 duplication that did not explain all of his clinical findings. The detection of *ANKRD11* alteration on chromosome 16q24.3, as determined through exome sequencing, provided a plausible explanation for most of the patient’s symptoms. The patient’s *CDH23* abnormality is the likely cause of his deafness. This case illustrates the wide diversity of clinical presentations of KBG syndrome and adds to the nascent body of literature on this topic. Our patient presents a case supporting the emerging evidence of a molecular basis between KBG syndrome and movement disorders. This report underscores the utility of exome sequencing as a first- or second-tier diagnostic tool in pediatric neurological and neurodevelopmental conditions. WES enhances our understanding of clinical presentations and diagnoses, especially among pediatric populations with neurodevelopmental disorders. By expediting diagnoses, WES minimizes the diagnostic odyssey for patients, facilitating access to needed resources and treatments. We put forth recommendations to expand access to WES for use in standard practice as a means for achieving improved long-term outcomes for patients and families.

## Acknowledgments

The authors would like to thank Dr Julie Beasley, the neuropsychologist who evaluated our patient, for her contributions to the clinical assessment. We are also grateful to Dr Kavita Batra, whose expertise as a biostatistician provided valuable guidance in shaping the direction of the project.

## Author contributions

**Conceptualization:** Elizabeth Wilkes, Holly N. Summers, Colleen A. Morris, Rooman F. Ahad.

**Data curation:** Elizabeth Wilkes, Holly N. Summers, Rooman F. Ahad.

**Formal analysis:** Holly N. Summers, Colleen A. Morris.

**Investigation:** Christina R. Dantam, Colleen A. Morris, Rooman F. Ahad.

**Methodology:** Elizabeth Wilkes.

**Project administration:** Colleen A. Morris, Rooman F. Ahad.

**Resources:** Christina R. Dantam, Elizabeth Wilkes, Holly N. Summers, Colleen A. Morris, Rooman F. Ahad.

**Supervision:** Colleen A. Morris, Rooman F. Ahad.

**Validation:** Colleen A. Morris.

**Writing – original draft:** Christina R. Dantam, Elizabeth Wilkes, Holly N. Summers, Colleen A. Morris, Rooman F. Ahad.

**Writing – review & editing:** Christina R. Dantam, Holly N. Summers, Colleen A. Morris, Rooman F. Ahad.

## References

[1] Herrmann J Pallister PD Tiddy W Opitz JM. The KBG syndrome-a syndrome of short stature, characteristic facies, mental retardation, macrodontia and skeletal anomalies. Birth Defects Orig Artic Ser. 1975;11:7–18. 1218237

[2] Martinez-Cayuelas E Blanco-Kelly F Lopez-Grondona F. Clinical description, molecular delineation and genotype–phenotype correlation in 340 patients with KBG syndrome: addition of 67 new patients. J Med Genet. 2023;60:644–54. 36446582 10.1136/jmg-2022-108632

[3] Peluso F Caraffi SG Contrò G. Deep phenotyping of the neuroimaging and skeletal features in KBG syndrome: a study of 53 patients and review of the literature. J Med Genet. 2023;60:1224–34. 37586838 10.1136/jmg-2023-109141 PMC10715526

[4] Gnazzo M Lepri FR Dentici ML. KBG syndrome: common and uncommon clinical features based on 31 new patients. Am J Med Genet A. 2020;182:1073–83. 32124548 10.1002/ajmg.a.61524

[5] Bayley N. Bayley Scales of Infant and Toddler Development: Administration Manual. 3rd ed. Pearson Assessments; 2006.

[6] Bayley N Aylward GP. Bayley Scales of Infant and Toddler Development: Administration Manual. 4th ed. Pearson Assessments; 2019.

[7] Alpern GD. Developmental Profile 4, DP-4: Manual. Western Psychological Services; 2020.

[8] Sparrow SS Cicchetti D Balla DA. Vineland Adaptive Behavior Scales (Vineland-II). 2nd ed. APA PsycTests; 2005.

[9] Sparrow SS Cicchetti D Saulnier CA. Vineland Adaptive Behavior Scales (Vineland-3). 3rd ed. APA PsycTests; 2016.

[10] Schopler E Van Bourgondien ME Wellman GJ Love SR. Childhood Autism Rating Scale. 2nd ed. Western Psychological Services; 2010.

[11] Lord C Rutter M DiLavore PC Risi S Gotham K Bishop S. Autism Diagnostic Observation Schedule. 2nd ed. Western Psychological Services; 2012.

[12] Donnellan EP Gorman KM Shahwan A Allen NM. Epileptic dyskinetic encephalopathy in KBG syndrome: expansion of the phenotype. Epilepsy Behav Rep. 2024;25:100647. 38317675 10.1016/j.ebr.2024.100647 PMC10839861

[13] Magistrelli L Contaldi E Caushi F. A case of early-onset Parkinson’s disease in a patient with KBG syndrome. Neurol Sci. 2023;44:4537–9. 37540342 10.1007/s10072-023-06988-2

[14] Thomsen M Lange LM Zech M Lohmann K. Genetics and pathogenesis of dystonia. Annu Rev Pathol. 2024;19:99–131. 37738511 10.1146/annurev-pathmechdis-051122-110756

[15] Oguro-Ando A Zuko A Kleijer KTE Burbach JPH. A current view on contactin-4, -5, and -6: implications in neurodevelopmental disorders. Mol Cell Neurosci. 2017;81:72–83. 28064060 10.1016/j.mcn.2016.12.004

[16] Wechsler D. Wechsler Abbreviated Scale of Intelligence--Second Edition. 2011.

[17] Hu J Liao J Sathanoori M. CNTN6 copy number variations in 14 patients: a possible candidate gene for neurodevelopmental and neuropsychiatric disorders. J Neurodev Disord. 2015;7:26. 26257835 10.1186/s11689-015-9122-9 PMC4528395

[18] Repnikova EA Lyalin DA McDonald K. CNTN6 copy number variations: uncertain clinical significance in individuals with neurodevelopmental disorders. Eur J Med Genet. 2020;63:103636. 30836150 10.1016/j.ejmg.2019.02.008

[19] Guo H Xun G Peng Y. Disruption of Contactin 4 in two subjects with autism in Chinese population. Gene. 2012;505:201–5. 22750301 10.1016/j.gene.2012.06.051

[20] Kashevarova AA Nazarenko LP Schultz-Pedersen S. Single gene microdeletions and microduplication of 3p26.3 in three unrelated families: CNTN6 as a new candidate gene for intellectual disability. Mol Cytogenet. 2014;7:97. 25606055 10.1186/s13039-014-0097-0 PMC4299808

[21] Tassano E Uccella S Giacomini T. Clinical and molecular characterization of two patients with CNTN6 copy number variations. Cytogenet Genome Res. 2018;156:144–9. 30508811 10.1159/000494152

[22] Rhamati L Marcolla A Guerrot AM. Audiological phenotyping evaluation in KBG syndrome: description of a multicenter review. Int J Pediatr Otorhinolaryngol. 2023;171:111606. 37336020 10.1016/j.ijporl.2023.111606

[23] Usami S Isaka Y Miyagawa M Nishio S. Variants in CDH23 cause a broad spectrum of hearing loss: from non-syndromic to syndromic hearing loss as well as from congenital to age-related hearing loss. Hum Genet. 2022;141:903–14. 35020051 10.1007/s00439-022-02431-2 PMC9034991

[24] Nolan D Carlson M. Whole exome sequencing in pediatric neurology patients: clinical implications and estimated cost analysis. J Child Neurol. 2016;31:887–94. 26863999 10.1177/0883073815627880

[25] Manickam K McClain MR Demmer LA; ACMG Board of Directors. Exome and genome sequencing for pediatric patients with congenital anomalies or intellectual disability: an evidence-based clinical guideline of the American College of Medical Genetics and Genomics (ACMG). Genet Med. 2021;23:2029–37. 34211152 10.1038/s41436-021-01242-6

[26] Hong C Wang J Xing C Hwang TH Park JY. Intersection of DNA privacy and whole-genome sequencing. Clin Chem. 2015;61:900–2. 25655271 10.1373/clinchem.2014.235499

